# PPAR-*γ* Mediates Ta-VNS-Induced Angiogenesis and Subsequent Functional Recovery after Experimental Stroke in Rats

**DOI:** 10.1155/2020/8163789

**Published:** 2020-07-22

**Authors:** Jiani Li, Keming Zhang, Qinbin Zhang, Xueling Zhou, Lan Wen, Jingxi Ma, Lingchuan Niu, Changqing Li

**Affiliations:** ^1^Department of Neurology, The Second Affiliated Hospital of Chongqing Medical University, Chongqing, China; ^2^Clinical College, Chongqing Medical and Pharmaceutical College, China; ^3^Department of Neurology, The First Affiliated Hospital of Chongqing Medical University, Chongqing, China; ^4^Department of Neurology, West China Hospital of Sichuan University, Sichuan, China; ^5^Department of Neurology, Chongqing General Hospital, University of Chinese Academy of Sciences, Chongqing, China; ^6^Chongqing Key Laboratory of Neurodegenerative Disease, Chongqing, China

## Abstract

**Background:**

Neoangiogenesis after cerebral ischemia in mammals is insufficient to restore neurological function, illustrating the need to design better strategies for improving outcomes. Our previous study has suggested that transcutaneous auricular vagus nerve stimulation (ta-VNS) induced angiogenesis and improved neurological functions in a rat model of cerebral ischemia/reperfusion (I/R) injury. However, the mechanisms involved need further exploration. Peroxisome proliferator-activated receptor-*γ* (PPAR-*γ*), well known as a ligand-modulated nuclear transcription factor, plays a crucial role in the regulation of cerebrovascular structure and function. Hence, the present study was designed to explore the role of PPAR-*γ* in ta-VNS-mediated angiogenesis and uncover the possible molecular mechanisms against ischemic stroke.

**Methods:**

Adult male Sprague–Dawley rats were transfected with either PPAR-*γ* small interfering RNA (siRNA) or lentiviral vector without siRNA prior to surgery and subsequently received ta-VNS treatment. The expression and localization of PPAR-*γ* in the ischemic boundary after ta-VNS treatment were examined. Subsequently, neurological deficit scores, neuronal damage, and infarct volume were all evaluated. Additionally, microvessel density, endothelial cell proliferation condition, and the expression of angiogenesis-related molecules in the peri-infarct cortex were measured.

**Results:**

We found that the expression of PPAR-*γ* in the peri-infarct cortex increased at 14 d and reached normal levels at 28 d after reperfusion. Ta-VNS treatment further upregulated PPAR-*γ* expression in the ischemic cortex. PPAR-*γ* was mainly expressed in neurons and astrocytes. Furthermore, ta-VNS-treated I/R rats showed better neurobehavioral recovery, alleviated neuronal injury, reduced infarct volume, and increased angiogenesis, as indicated by the elevated levels of brain-derived neurotrophic factor (BDNF), vascular endothelial growth factor (VEGF), and phosphorylated endothelial nitric oxide synthase (P-eNOS). Surprisingly, the beneficial effects of ta-VNS were weakened after PPAR-*γ* silencing.

**Conclusions:**

Our results suggest that PPAR-*γ* is a potential mediator of ta-VNS-induced angiogenesis and neuroprotection against cerebral I/R injury.

## 1. Background

Ischemic stroke continues to be a serious worldwide health problem that leads to a high level of disability and mortality [1]. Ischemia results from the obstruction of cerebral blood flow. In response to the loss of blood supply, the body undergoes angiogenesis, the formation of new blood vessels from preexisting vessels. Recent studies have suggested that angiogenesis not only provides sufficient oxygen and nutrients but also offers a niche for the survival of neurons after cerebral ischemia/reperfusion (I/R) injury [2–4]. More importantly, the increased angiogenesis is often closely associated with reduced cerebral infarction and improved neurofunctional recovery. Therefore, to enhance angiogenesis and rescue damaged neurons are considered effective therapeutic strategies for cerebral I/R injury.

Peroxisome proliferator-activated receptor *γ* (PPAR-*γ*) is a multifunctional ligand-activated nuclear transcription factor that has been found to play crucial roles in the regulation of angiogenesis as well as confer significant neuroprotection against central nervous system (CNS) injury [5, 6]. It has also been shown to promote the synthesis and secretion of angiogenic factors [7, 8]. In addition, a previous study demonstrated that PPAR-*γ* agonist, rosiglitazone (RGZ), promoted angiogenesis and neurofunctional recovery after cerebral ischemia [9]. Although the PPAR-*γ* agonists can exert important proangiogenic protection, the compounds also have side effects, including fluid retention, weight gain, and the risk of heart attack [10]. Therefore, developing new clinical approach that is both effective and safe is urgently needed for the treatment of ischemic stroke.

Transcutaneous electrical stimulation of the auricular branch of the vagus nerve (ta-VNS) has been proved to be an experimental therapeutic method to exert neuroprotective effects following cerebral ischemia. Our previous studies showed that ta-VNS treatment reduced infarct volume, promoted angiogenesis, and subsequent functional recovery in rat models of middle cerebral artery occlusion/reperfusion (MCAO/R) [11, 12]. Most recently, it has been demonstrated that ta-VNS activated the vagal pathway and exerted neuroprotection similar to that of the traditional cervical vagus nerve stimulation (c-VNS) [13]. Therefore, it is a novel and noninvasive treatment for ischemic stroke. However, the mechanisms of ta-VNS-induced neovascularization are not fully understood. Owing to the important role of PPAR-*γ* on cerebral ischemia, the present study was designed to investigate whether PPAR-*γ* was involved in the proangiogenic activity induced by ta-VNS and its mechanism after ischemic brain injury.

## 2. Methods

### 2.1. Animals and Experimental Design

Adult male Sprague-Dawley (SD) rats (250-350 g) were obtained from the Experimental Animal Center of Chongqing Medical University and housed in a quiet room maintained at 21–22°C (60% humidity on a 12 h light/12 h dark cycle), with food and water freely available throughout the experiment. All animal procedures were approved by the Institutional Ethics Committee of Chongqing Medical University and performed strictly in accordance to the Guidelines for the Care and Use of Laboratory Animals. There were two parts in the experiment, and the rats were randomly assigned to the experimental groups. To investigate PPAR-*γ* expression in the peri-infarct cortex, the first experiment was divided into 4 groups (*n* = 8/group): (1) sham group, (2) I/R group, (3) I/R with sham stimulation (IR+SS group), and (4) I/R with ta-VNS group (IR+ta-VNS group). To explore the role of PPAR-*γ* in ta-VNS-mediated angiogenesis, the second part included 4 groups (*n* = 10/group): (1) sham+LV-control group, (2) I/R+LV-control group, (3) I/R+ta-VNS+LV-control group, and (4) I/R+ta-VNS+LV-siPPAR-*γ* group.

### 2.2. Lentivirus Injection In Vivo

The pHBLV-U6-MCS-PGK-PURO small interfering RNA (siRNA) vector was constructed by a reagent company (Hanbio Tech, Shanghai, China) with the sequence CCACACTATGAAGACATCCCGTTCA. Fourteen days before the cerebral I/R injury, lentiviruses were stereotaxically injected into the ischemic cortex. The coordinates in the right cortex were as follows: AP: -3.0 mm, ML: -1.5 mm, DV: -1.2 mm; AP: 1.0 mm, ML: -2.0 mm, and DV: -1.2 mm. The injection rate was 0.3 *μ*l/min, and the volume was 2.5 *μ*l for each site. By the end of the injection, the microinjector was left for an additional 5 min before withdrawal.

### 2.3. Build a Model of MCAO/R and Ta-VNS Treatment

Right middle cerebral artery occlusion/reperfusion (MCAO/R) model was performed as previously described [14]. Briefly, rats were anesthetized with phenobarbital sodium (60 mg/kg, intraperitoneal injection). The right common carotid artery, external carotid artery (ECA), and internal carotid artery (ICA) were sequentially exposed. A nylon filament with a blunted tip was advanced from the ECA into the ICA. The filament was withdrawn for blood reperfusion after 2 h of occlusion. To confirm the success of the MCAO/R model, cerebral blood flow changes were continuously monitored by laser Doppler flowmetry (LDF) during occlusion and early reperfusion [15]. Rats with an occluded blood flow reduction of less than 70% or a rapid restoration of the LDF signal during reperfusion were excluded from the study. The tail arterial pressure, blood gas levels, and heart rate were also measured during the experimental process and the body temperature was maintained at approximately 37°C with an electrothermal pad [16]. According to our previous study [12], rats received ta-VNS with 2 acupuncture needles connected to a Grass Model S48 stimulator and the needles were inserted 0.5–1 mm under the skin over the left cavum concha. The stimulation parameters were 0.5 ms square pulses lasting 30 s (0.5 mA) at 20 Hz and repeated every 5 min for 1 h. The animals received ta-VNS treatment twice daily starting 30 min after MCAO until the time of sacrifice. The same operation was performed on the I/R+SS group without stimulation.

### 2.4. Neurobehavioral Evaluation

The modified neurological severity scores (mNSS) and adhesive-removal somatosensory tests were performed before surgery (baseline) as well as 14 and 28 d after MCAO/R.

The mNSS is a composite test [17], including sensory, motor, balance, and reflect. Neurological function score was from 0 to 18 (normal score—0; maximal deficit score—18). In the severity scores of injury, 1 point represents the inability to complete the test or the lack of a tested reflex. Therefore, if the score was higher, the more severe the injury would be.

The adhesive-removal somatosensory test was used to assess sensorimotor deficits [18]. Two pieces of circular adhesive-backed papers (area: 113.1 mm^2^) were applied as bilateral tactile stimuli occupying the distal-radial region of each forelimb. The time that moved each stimulus was recorded during 3 tests every day (maximum time limit—120 s). Individual tests were separated by at least 10 min. The animals would be pretrained for 3 d in order to decrease individual differences before the surgery.

### 2.5. Cerebral Infact Volume Measurement

The rats were deeply anesthetized using phenobarbital sodium (60 mg/kg, intraperitoneal injection) and then decapitated at 28 d after reperfusion, and then, their brain tissues were cut into 2 mm thick sections and immersed with 2% 2,3,5-triphenyltetrazolium chloride (37°C for 30 min) followed by fixation with 4% paraformaldehyde. The stained sections were photographed, and the infarct area was calculated by Image-Pro Plus 6.0 software. The infarct volumes were measured as previously described [19].

### 2.6. Brain Nissl Staining

After four weeks of MCAO/R, the animals were sacrificed. The brains were buried in paraffin and cut into 4 *μ*m thick sections. Then, the sections were processed for nissl staining [20]. Histological changes of ischemic cortex were observed by the microscope (original magnification x400) to assess brain neuronal damage.

### 2.7. Real-Time PCR

Total RNA of the right ischemic cortex was extracted for the assay of PPAR-*γ*, BDNF, and VEGF mRNA levels after I/R injury. The procedure was similar to that in our previous study [21]. The following primers were used for amplification. PPAR-*γ*: forward 5′-CACAATGCCATCAGGTTTGG-3′ and reverse 5′-GCTGGTCGATATCACTGGAGATG-3′; BDNF: forward 5′-TGTCCGAGGTGGTAGTACTTCATC-3′ and reverse 5′-CATGCAACCGAAGTATGAAATAACC-3′; VEGF: forward 5′-GCGGGCTGCCTCGCAGTC-3′ and reverse 5′-TCACCGCCTTGGCTTGTCAC-3′; *β*-actin: forward 5′-CGTTGACATCCGTAAAGACCTC-3′ and reverse 5′-TAGGAGCCAGGGCAGTAATCT-3′. The relative gene expression was calculated using the 2^−*ΔΔ*CT^ method [22].

### 2.8. Western Blotting

Proteins of different groups in the peri-infarct cortex were collected after MCAO/R. Western blotting was processed according to the standard protocols. Briefly, the proteins were electrophoresed with SDS polyacrylamide gel and transferred onto PVDF membrane (Millipore, USA). The membranes were incubated with the following primary antibodies: anti-PPAR-*γ* (1 : 800, Abcam), anti-BDNF (1 : 500, Abcam), anti-VEGF (1 : 800, Proteintech), and anti-GAPDH (1 : 4000, Proteintech). After washing with PBS, the membrane was incubated with peroxidase-conjugated secondary antibodies. The images of western blotting were captured and quantified using the Fusion FX5 analysis system.

### 2.9. ELISA

Ischemic cortex tissues were harvested for the assay of p-eNOS levels at 28 d after I/R injury. The expression of p-eNOS in the tissue homogenates was measured using a rat p-eNOS ELISA kit, according to the manufacturers' protocols (Hushang, Shanghai, China).

### 2.10. Immunofluorescence

The brain tissues of sacrificed rats at 28 d after reperfusion were fixed with 4% paraformaldehyde, cut into coronal sections, permeabilized with 0.4% Triton X-100, and stained overnight at 4°C with primary antibodies diluted in PBS.(anti-PPAR-*γ* (1 : 50, Abcam), anti-NeuN (1 : 200, Millipore), anti-GFAP (1 : 100, Boster), anti-CD31 (1 : 50, R&D systems), anti-Ki67 (1 : 50, Abcam), and anti-FLAG (1 : 50, Proteintech)). The next day, the sections were incubated with secondary antibodies for 1 h at room temperature. After washing, the sections were counterstained with DAPI for nuclei. Then, images were captured by a light microscope (Olympus DP70, Japan) and quantified using Image-Pro Plus 6.0 software.

### 2.11. Statistical Analysis

The data are expressed as means ± SD. GraphPad Prism 8.0 was used for graphing and statistical analysis. One-way or two-way analysis of variance (ANOVA) followed by Tukey's post hoc multiple comparison test was utilized to compare the differences between groups. *P* < 0.05 was regarded statistically significant.

## 3. Results

### 3.1. Physiological Parameters

Physiological parameters including blood pressure, blood gases, and heart rate were all within normal range between the groups (Table 1), which was consistent with our previous study [16].

### 3.2. PPAR-*γ* Expression in the Peri-Infarct Cortex Is Enhanced after Ta-VNS Treatment

**Figure 1:**
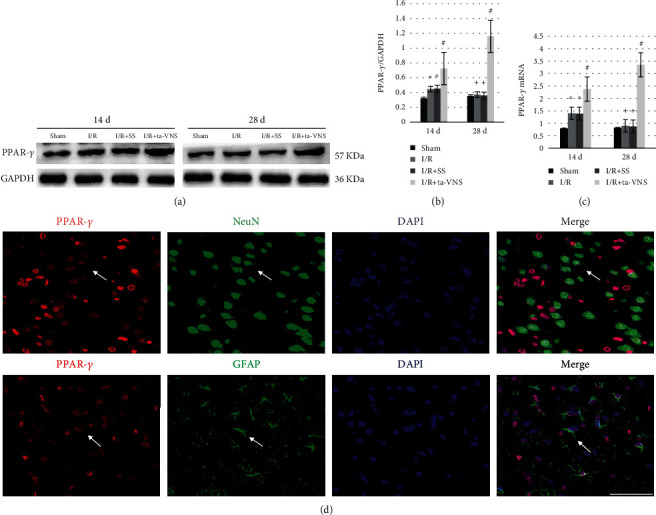
PPAR-*γ* expression in the peri-infarct cortex is upregulated by ta-VNS treatment. (a) Representative western blot images of PPAR-*γ* expression induced by ta-VNS at 14 and 28 d after MCAO/R. (b) Comparison of mean intensity ratios in western blot analysis for PPAR-*γ* expression.(^∗^*P* < 0.05 vs sham group, ^#^*P* < 0.05 vs I/R and I/R+SS groups, ^+^*P* > 0.05 vs sham group). (c) qPCR analysis showing PPAR-*γ* gene expression induced by ta-VNS at 14 and 28 d after MCAO/R. (^∗^*P* < 0.05 vs sham group, ^#^*P* < 0.05 vs I/R and I/R+SS groups, ^+^*P* > 0.05 vs sham group). (d) Immunofluorescence staining for PPAR-*γ* (red), GFAP/NeuN (green), and cellular nuclei (blue) at 28 d after reperfusion. Arrows show the positive cells. Scale bar = 50 *μ*m.

To explore the possible relationship between the ta-VNS and PPAR-*γ*, the expression and location of PPAR-*γ* in the peri-infarct cortex were examined by western blotting, qPCR, and immunofluorescence. Western blotting and qPCR showed that the mRNA and protein levels of PPAR-*γ* were higher at 14 d and gradually decreased to a normal level at 28 d in the ischemic cortex after I/R injury, and the expression was further elevated by ta-VNS treatment at both time points (Figures 1(a)-1(c)). Moreover, PPAR-*γ* was expressed in neurons and astrocytes (Figure 1(d)). These data suggested that upregulation of PPAR-*γ* expression could be induced by ta-VNS treatment.

### 3.3. Expression of PPAR-*γ* after Injection of Recombinant Lentivirus

**Figure 2:**
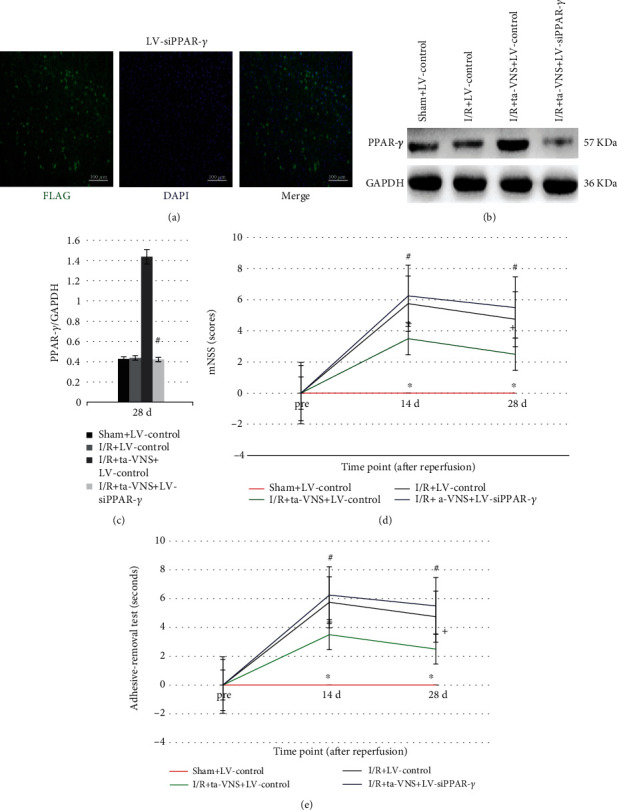
Lentiviruses were successfully transfected into neurons and reduced the expression of PPAR-*γ*; the neuroprotective effect induced by ta-VNS is reduced by PPAR-r silencing. (a) Fluorescent images of FLAG (green) in the right cortex 14 d after injection (scale bar = 100 *μ*m). (b, c) Western blot images and statistical histogram of PPAR-*γ* expression with LV-siPPAR-*γ* injection 28 d after MCAO/R. ^∗^*P* < 0.05, compared to the I/R+LV-control group. (d, e) The mNSS and adhesive-removal somatosensory tests were performed before surgery and 14 and 28 d after MCAO/R (^+^*P* < 0.05 vs sham+LV-control group, ^∗^*P* < 0.05 vs I/R+LV-control group, and ^#^*P* < 0.05 vs I/R+ta-VNS+LV-control group).

LV-siPPAR-*γ* or LV-control were injected into the ischemic cortex 14 d before MCAO/R. Flag protein carried by lentivirus was observed in the injected cortex 14 d after injection (Figure 2(a)). The expression of PPAR-*γ* was significantly lower in the I/R+ta-VNS+LV-siPPAR-*γ* group than that in the I/R+ta-VNS+LV-control group at 28 d after MCAO/R (*P* < 0.05, Figures 2(b) and 2(c)), indicating that LV-siPPAR-*γ* efficiently reduced the expression of PPAR-*γ*.

### 3.4. Inhibition of PPAR-*γ* Weakens the Improvements on Neurological Scores Induced by Ta-VNS after I/R Injury

To identify the effects of PPAR-*γ* on neurological behavior impairment under cerebral I/R injury and ta-VNS treatment, the mNSS and adhesive-removal somatosensory tests were performed to evaluate neurological function 3 d before and 14 and 28 d after MCAO/R. Before surgery, no significant differences in the neurological scores were detected among the groups in both tests. Rats in the I/R group exhibited sustained neurobehavioral impairments as compared to the sham group in both tests, indicating that cerebral ischemia could induce neurological deficits. Ta-VNS treatment prevented neurological impairment, and these effects were also observed in the I/R+ta-VNS+LV-control group, which is consistent with our previous studies [11, 12]. Nevertheless, the beneficial effects of ta-VNS were abrogated after PPAR-*γ* silencing (*P* < 0.05, Figures 2(d) and 2(e)). These data indicated that PPAR-*γ* can protect the brain against transient cerebral I/R injury and mediate the neuroprotective effects induced by ta-VNS during the chronic stage of ischemic stroke.

### 3.5. Inhibition of PPAR-*γ* Attenuates the Ta-VNS-Mediated Suppression of Neuronal Damage and Infarction Volume Enlargement after I/R Injury

**Figure 3:**
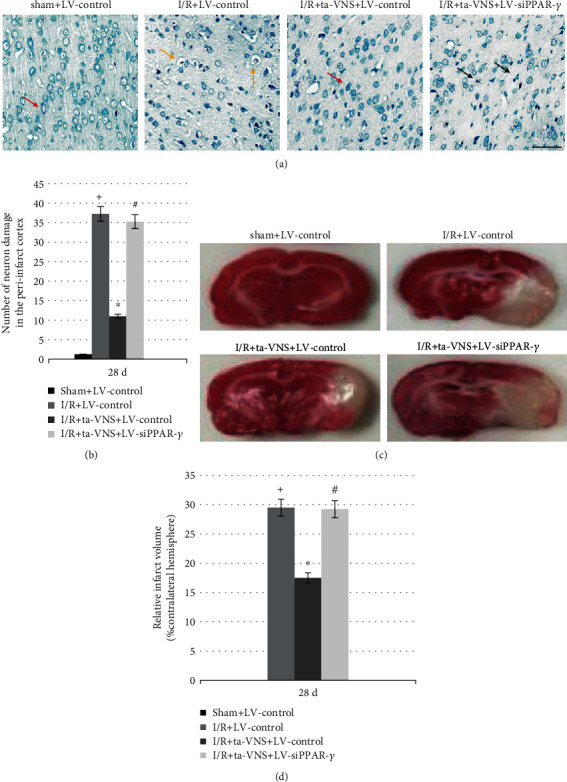
Inhibition of PPAR-*γ* attenuates the ta-VNS-mediated suppression of neuronal damage and infarction volume enlargement at 28 d after MCAO/R. (a) Representative images of nissl staining results in each group (scale bar = 50 *μ*m). Red arrows represent normal neuron, yellow arrows represent perinuclear vacuolization, and black arrows represent pyknotic nuclei. (b) The number of neuron damage in the peri-infarct cortex was represented in the bar graph. (c) Representative images of infarction volume in each group. (d) The measure of infarct volume was reflected in the bar graph at 28 d after stroke (^+^*P* < 0.05 vs sham+LV-control group, ^∗^*P* < 0.05 vs I/R+LV-control group, and ^#^*P* < 0.05 vs I/R+ta-VNS+LV-control group).

To further explore the role of PPAR-*γ* on the neuroprotective effects induced by ta-VNS under cerebral ischemia, we evaluated the nissl staining and infarct volume in rats at 28 d after reperfusion. As shown in Figure 3, in the sham group, the neurons had a normal cellular morphology. However, ischemia caused neuron damage in the ischemic cortex, which exhibited vacuolization, disordered structure, reduced nissl bodies, and severe cell deformation with karyopyknotic nuclei. Compared with the I/R group, the number of neuronal damage after ta-VNS treatment (I/R+ta-VNS+LV-control group) appeared to be reduced, with a greater density of surviving neurons and relatively intact cellular structure. Nevertheless, the neuroprotection was abrogated in the I/R+ta-VNS+LV-siPPAR-*γ* group compared with the I/R+ta-VNS+LV-control group (*P* < 0.05). The changes of infarction volume in each group were consistent with the results of neuronal damage.

The infarction volume was similarly increased after reperfusion compared with the sham group, and this increase was abrogated by ta-VNS treatment (*P* < 0.05) but dramatically suppressed by LV-siPPAR-*γ* treatment (*P* < 0.05). These results showed that PPAR-*γ* may play a crucial role in the neuroprotective effect of ta-VNS following I/R injury.

### 3.6. Inhibition of PPAR-*γ* Attenuates the Ta-VNS-Mediated Promotion of Angiogenesis after I/R Injury

**Figure 4:**
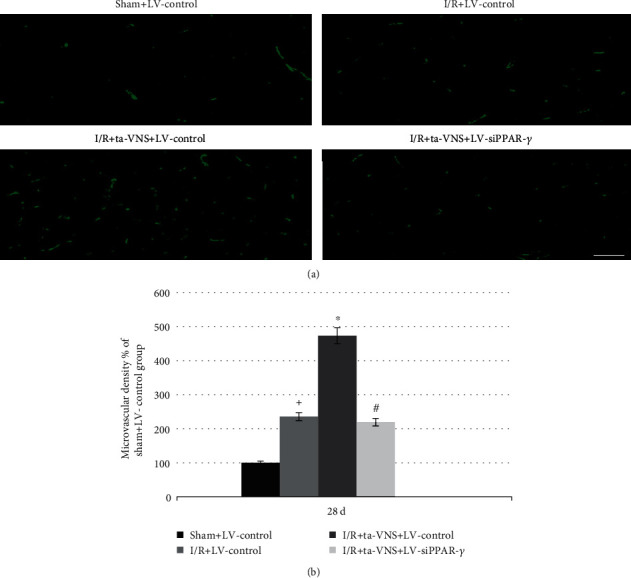
Inhibition of PPAR-*γ* attenuates the ta-VNS-mediated increase of cerebral microvessel density at 28 d after MCAO/R. (a) CD31 staining shows the cerebral microvessel density around the postinfarct area (scale bar = 50 *μ*m). (b) Statistical comparison of the microvessel density in each group (^+^*P* < 0.05 vs sham+LV-control group, ^∗^*P* < 0.05 vs I/R+LV-control group, and ^#^*P* < 0.05 vs I/R+ta-VNS+LV-control group).

**Figure 5:**
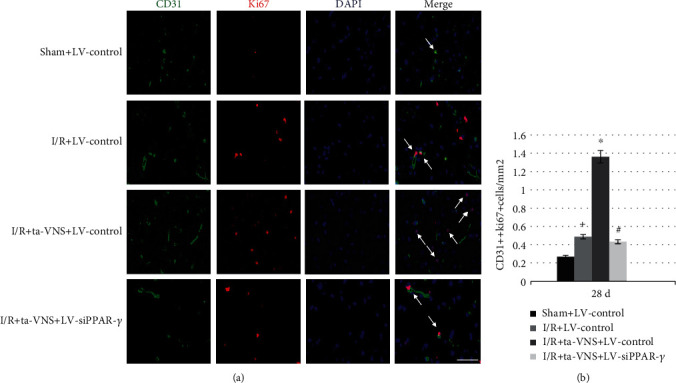
Inhibition of PPAR-*γ* attenuates the ta-VNS-mediated promotion of endothelial cell proliferation at 28 d after MCAO/R. (a) The figure shows ki67 (red) and CD31 (green) double staining in each group. Arrows show the colocalization of CD31 and Ki67 (scale bar = 50 *μ*m). (b) Statistical comparison of the proliferating endothelial cells in each group (^+^*P* < 0.05 vs sham+LV-control group, ^∗^*P* < 0.05 vs I/R+LV-control group, and ^#^*P* < 0.05 vs I/R+ta-VNS+LV-control group).

To further investigate the role of PPAR-*γ* in the ta-VNS-induced angiogenesis, we examined the microvessel density and proliferating endothelial cells (ECs) around the ischemic lesion. Anti-CD31 was used to detect ECs, and anti-ki67 was used to detect proliferating cells. At 28 d after MCAO/R, the number of microvessels with staining of CD31 and proliferating ECs labeled with double staining of CD31 and ki67 in the peri-infarct cortex was increased in the I/R group compared with that of the sham group. The results were further increased after ta-VNS treatment; nevertheless, this increase was abrogated in the I/R+ta-VNS+LV-siPPAR-*γ* group compared with the I/R+ta-VNS+LV-control group (*P* < 0.05, Figures 4(a) and 4(b) and 5(a) and 5(b)). Our results indicated that PPAR-*γ* appears to be a proangiogenic factor against ischemic stroke and may mediate the promotion of angiogenesis induced by ta-VNS in the ischemic boundary during the recovery period.

### 3.7. Inhibition of PPAR-*γ* Attenuates the Ta-VNS-Mediated Promotion of Brain-Derived Neurotrophic Factor (BDNF), Vascular Endothelial Growth Factor (VEGF), and Phosphorylated Endothelial Nitric Oxide Synthase (P-eNOS) Expression Induced by Ta-VNS after I/R Injury

**Figure 6:**
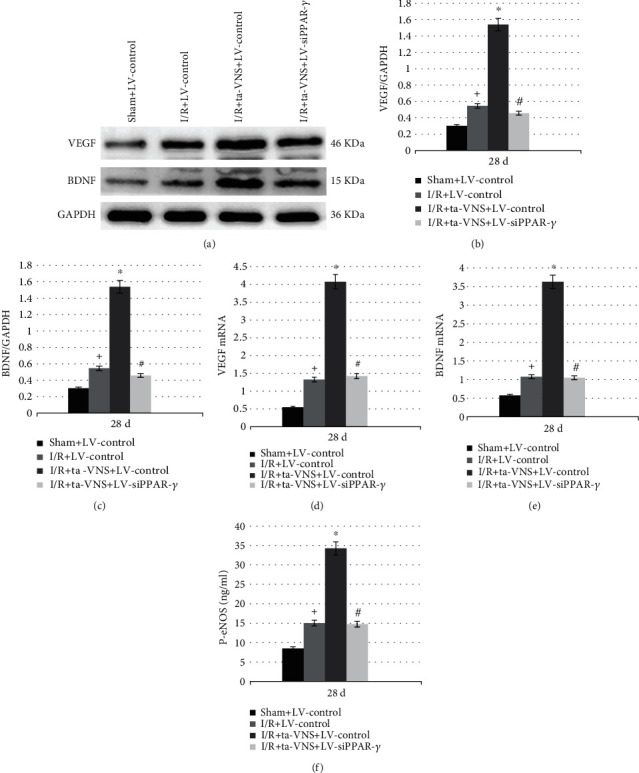
Inhibition of PPAR-*γ* suppresses the ta-VNS-mediated promotion of BDNF, VEGF, and P-eNOS expression at 28 d after MCAO/R. (a) Representative western blot images of VEGF and BDNF expression in the peri-infarct cortex. (b, c) Quantitative results from (a). (d) The bar graph reflected VEGF mRNA expression in each group. (e) The bar graph reflected BDNF mRNA expression in each group. (f) ELISA assay for P-eNOS protein expression (^+^*P* < 0.05 vs sham+LV-control group, ^∗^*P* < 0.05 vs I/R group+LV-control, and ^#^*P* < 0.05 vs I/R+ta-VNS+LV-control group).

To explore the mechanism of PPAR-*γ* in the proangiogenic effect induced by ta-VNS after MCAO/R, the expression levels of BDNF, VEGF, and p-eNOS at 28 d after reperfusion were tested. Compared with the sham group, the mRNA and protein levels of BDNF and VEGF were significantly increased in the I/R+LV-control group, and the levels were further enhanced in the I/R+ta-VNS+LV-control group compared with the I/R+LV-control group, whereas the upregulation was diminished in the I/R+ta-VNS+LV-siPPAR-*γ* group relative to the I/R+ta-VNS+LV-control group (*P* < 0.05, Figures 6(a)-6(e)). The ELISA analysis of p-eNOS levels was consistent with the BDNF and VEGF expressions (*P* < 0.05, Figure 6(f)). Based on these data, we speculated that PPAR-*γ* upregulation was involved in the promotion of BDNF, VEGF, and p-eNOS expressions induced by ta-VNS after MCAO/R.

## 4. Discussion

In our study, we performed a series of experiments indicating that ta-VNS was capable of promoting angiogenesis and improving neurofunctional recovery at the chronic stage of ischemic stroke. Moreover, our findings also revealed that PPAR-*γ* might be involved in promoting angiogenesis induced by ta-VNS after acute cerebral I/R injury, which represents an important potential target for ischemic stroke.

PPAR-*γ* is a widely expressed ligand-modulated nuclear transcription factor that governs the expression of genes involved in inflammation, redox equilibrium, and growth factor production. The expression level of PPAR-*γ* is relatively low in the normal adult brain, but the expression of PPAR-*γ* is markedly increased in the ischemic cerebral cortex after stroke [23, 24]. In line with these studies, in our study, the mRNA and protein levels of PPAR-*γ* were upregulated at 14 d and reached normal levels at 28 d after reperfusion, and both were further increased after ta-VNS treatment. In addition, PPAR-*γ* was observed coexpressing with neurons and astrocytes in the peri-infarct cortex, indicating that PPAR-*γ* participates in the pathological process of cerebral I/R injury. According to many studies [6, 10, 25], the upregulation and activation of PPAR-*γ* seem to confer protection in the acute phase of cerebral ischemic injury. In this study, PPAR-*γ* upregulation induced by ta-VNS showed reduced infarct volume, decreased neuron damage, and improved neurofunctional recovery during the recovery stage of ischemic stroke, while inhibition of PPAR-*γ* attenuated the ta-VNS-mediated neuroprotection. These data clearly indicate that PPAR-*γ* may be involved in the neuroprotection induced by ta-VNS in the chronic repair phase of ischemic stroke. However, more detailed mechanisms underlying the PPAR-*γ*-dependent neuroprotection need further exploration.

There is mounting evidence showing that neoangiogenesis serves as a natural process during the chronic phase of cerebral IR injury, which requires EC proliferation, differentiation, and migration to form new blood vessels in order to compensate the hypoxic environment and is closely associated with neurofunctional recovery [2, 3]. PPAR-*γ* is highly expressed in vascular walls, including the vascular smooth muscle cells and vascular ECs [26]. The importance of PPAR-*γ* in the angiogenesis after stroke was reported by Higgins et al. [10]; administration of PPAR-*γ* agonist RGZ to rats caused significant increases in EC proliferation, microvessel density, vascular branch points, and surface areas in the ischemic boundary zone. Another recent study demonstrated that the loss of PPAR-*γ* inhibited angiogenesis and migration capacity of human pulmonary microvascular EC [5], suggesting that PPAR-*γ* would be beneficial for angiogenesis. However, whether PPAR-*γ* is involved in the angiogenesis induced by ta-VNS after cerebral ischemia remains unclear.

Our study revealed that ta-VNS treatment significantly promoted EC proliferation and the increase of microvessel density around the post-infarct area; nevertheless, the proangiogenic effects induced by ta-VNS were diminished after downregulation of PPAR-*γ*. Therefore, our results indicated that PPAR-*γ* may serve as an important mediator involved in the process of ta-VNS-triggered angiogenesis and neuroprotection against ischemic stroke.

We further explored the potential molecular mechanism of PPAR-*γ* in angiogenesis induced by ta-VNS after cerebral I/R injury. Recently, it has been demonstrated that PPAR-*γ* might be involved in mediating growth factor production. Selective activation of PPAR-*γ* led to the upregulation of BDNF and VEGF expression [7, 8]. As we all know, VEGF is a potent angiogenic factor that is involved in the process of neoangiogenesis and has been studied extensively [27]. There is now ample evidence showing that the angiogenic effect of VEGF is mediated, in part, through P-eNOS which acts as a survival factor for endothelial cells by both inhibiting cell death and advancing endothelial cell migration and tube formation [28, 29]. eNOS overexpression enhanced angiogenic growth potential and vascular recovery in the ischemic retina [30]. Moreover, Wang et al. found that VEGF protected neurons from cell death directly rather than only by advancing angiogenesis [31]. Hence, VEGF is an important therapy target for ischemic stroke. In addition to VEGF, BDNF, as a novel angiogenic growth factor, plays a critical role in angiogenesis [32]. In one study, BDNF could act as a key inducer of angiogenesis and boost the proliferation of human umbilical vein ECs [33]. In a second study, downregulation of BDNF expression could impair the connections and survival of ECs [34]. Furthermore, BDNF upregulation was reported to enhance expression and secretion of VEGF and p-eNOS [35], indicating its potential molecular mechanism of inducing angiogenesis. However, whether ta-VNS could influence the expression of BDNF, VEGF, and p-eNOS through PPAR-*γ* in cerebral ischemia was unknown. As demonstrated in our study, we confirmed that the neoangiogenesis could be enhanced by ta-VNS. One of the possible mechanisms underlying the ta-VNS-induced neoangiogenesis is that PPAR-*γ*-dependent upregulation of BDNF, VEGF, and p-eNOS expression, which is known to be responsible for the promotion of angiogenesis in the CNS.

## 5. Conclusion

In conclusion, the results in our study indicate that PPAR-*γ* may be involved in the promotion of angiogenesis induced by ta-VNS against I/R injury. Downregulation of PPAR-*γ* attenuated the neoangiogenesis induced by ta-VNS after MCAO/R. Our data provide worthy insight into the correlation between PPAR-*γ* and ta-VNS-induced neuroprotection in the chronic phase of ischemic stroke.

## Figures and Tables

**Table 1:** The physiological parameters during the experiment (all data are shown as the mean ± SD).

Group	Mean blood pressure (mmHg)	Heart rate (bp/min)	pH	PCO_2_ (mmHg)	PO_2_ (mmHg)
Sham	83 ± 8.1	365 ± 8	7.37 ± 0.02	46.3 ± 0.9	115.3 ± 10.1
I/R	84 ± 7.2	364 ± 9	7.38 ± 0.01	44.7 ± 1.7	110.3 ± 11.2
I/R+SS	86 ± 6.1	361 ± 11	7.39 ± 0.01	45.6 ± 1.3	111.1 ± 11.3
I/R+ta-VNS	85 ± 6.6	360 ± 11	7.36 ± 0.02	45.1 ± 1.2	107.4 ± 12.1
Sham+LV-control	86 ± 6.4	363 ± 10	7.36 ± 0.03	46.6 ± 1.1	108.9 ± 12.3
I/R+LV-control	83 ± 7.8	362 ± 12	7.35 ± 0.03	45.9 ± 1.6	114.7 ± 9.8
I/R+ta-VNS+LV-control	87 ± 4.3	365 ± 9	7.36 ± 0.03	46.1 ± 1.0	114.2 ± 9.4
I/R+ta-VNS+LV-siPPAR-*γ*	86 ± 5.3	367 ± 8	7.37 ± 0.01	45.6 ± 1.3	109.7 ± 12.6

## Data Availability

The datasets used or analyzed during this study are available from the corresponding author on reasonable request.

## Additional Points

*Highlights*. (1) Repeated ta-VNS treatment promoted angiogenesis and improved long-term functional recovery following MCAO/R. (2) Ta-VNS-triggered neuroprotection was associated with PPAR-*γ* upregulation in the peri-infarct cortex. (3) Inhibition of PPAR-*γ* abolished ta-VNS-induced angiogenesis and neuroprotection following MCAO/R. (4) Ta-VNS treatment exerted proangiogenic effects via the promotion of BDNF, VEGF, and P-eNOS expression in a PPAR-*γ*-dependent manner.
