# Correction to “PPAR‐*γ* Mediates Ta‐VNS‐Induced Angiogenesis and Subsequent Functional Recovery after Experimental Stroke in Rats”

**DOI:** 10.1155/bmri/9783945

**Published:** 2026-07-15

J. Li, K. Zhang, Q. Zhang, et al., “PPAR‐*γ* Mediates Ta‐VNS‐Induced Angiogenesis and Subsequent Functional Recovery After Experimental Stroke in Rats,” BioMed Research International 2020 (2020): 8163789, https://doi.org/10.1155/2020/8163789.

In the above‐mentioned article, there were errors in Figures 2d,e and 5b.

The graphs in Figures 2d,e were inadvertently duplicated. The corrected figure panels are shown as follows:

Figure 2Lentiviruses were successfully transfected into neurons and reduced the expression of PPAR‐*γ*; the neuroprotective effect induced by ta‐VNS is reduced by PPAR‐r silencing. (a) Fluorescent images of FLAG (green) in the right cortex 14 d after injection (*s* *c* *a* *l* *e* *b* *a* *r* = 100 *μ* *m*). (b, c) Western blot images and statistical histogram of PPAR‐*γ* expression with LV‐siPPAR‐*γ* injection 28 d after MCAO/R.  ^∗^ *p* < 0.05, compared with the I/R + LV‐control group. (d, e) The mNSS and adhesive‐removal somatosensory tests were performed before surgery and 14 and 28 d after MCAO/R (^+^ *p* < 0.05 vs. *s* *h* *a* *m* + *L* *V* − *c* *o* *n* *t* *r* *o* *l* *g* *r* *o* *u* *p*,  ^∗^ *p* < 0.05 vs. *I*/*R* + *L* *V* − *c* *o* *n* *t* *r* *o* *l* *g* *r* *o* *u* *p*, and ^#^ *p* < 0.05 vs. *I*/*R* + *t* *a* − *V* *N* *S* + *L* *V* − *c* *o* *n* *t* *r* *o* *l* *g* *r* *o* *u* *p*).

**Figure:**
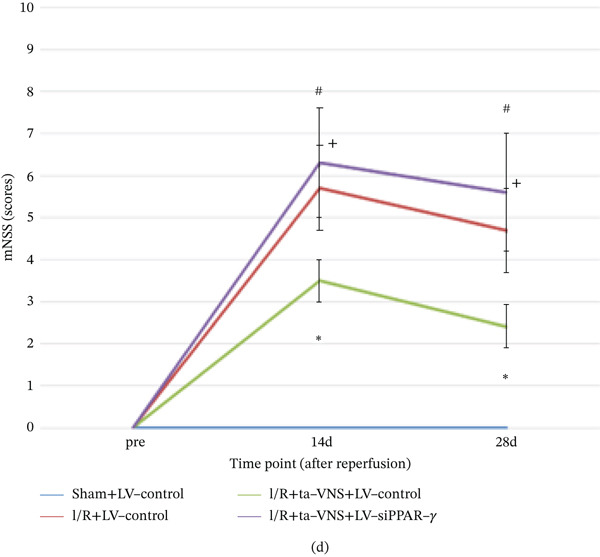
(d)

**Figure:**
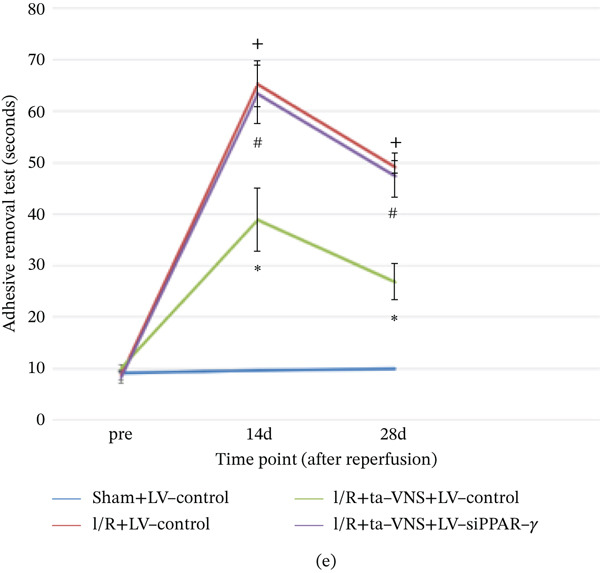
(e)

In Figure 5b, the incorrect unit measurements were included on the y‐axis. The corrected figure panel is shown below:

**Figure 5:**
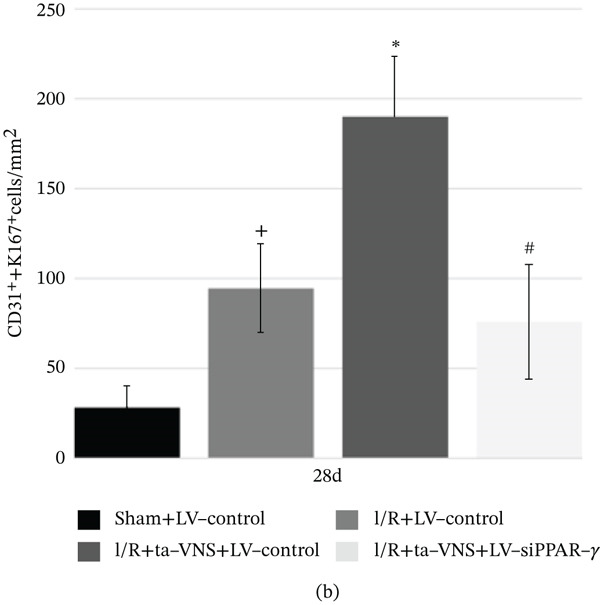
Inhibition of PPAR‐*γ* attenuates the ta‐VNS‐mediated promotion of endothelial cell proliferation at 28 d after MCAO/R. (a) The figure shows ki67 (red) and CD31 (green) double staining in each group. Arrows show the colocalization of CD31 and Ki67 (*s* *c* *a* *l* *e* *b* *a* *r* = 50 *μ* *m*). (b) Statistical comparison of the proliferating endothelial cells in each group (^+^ *p* < 0.05 vs. *s* *h* *a* *m* + *L* *V* − *c* *o* *n* *t* *r* *o* *l* *g* *r* *o* *u* *p*, ∗*p* < 0.05 vs. *I*/*R* + *L* *V* − *c* *o* *n* *t* *r* *o* *l* *g* *r* *o* *u* *p*, and ^#^ *p* < 0.05 vs. *I*/*R* + *t* *a* − *V* *N* *S* + *L* *V* − *c* *o* *n* *t* *r* *o* *l* *g* *r* *o* *u* *p*).

We apologize for these errors.

